# Supplementary material for: Content-rich biological network constructed by mining PubMed abstracts
Source: BMC Bioinformatics. 2004 Oct 8;5:147. doi: 10.1186/1471-2105-5-147 (PMC528731; doi:10.1186/1471-2105-5-147)
Supplement: Additional File 2 — The original results of the above study (non-essential files are deleted to keep the file size under the limit set by BMC bioinformatics). [file 1471-2105-5-147-S2.bz2 › chilibotAdditionalFile2/dip20/79ID9033409E291/html/CDC42_PAK3.html]

 


 **CDC42** and **PAK3** 
  
Found 226 abstracts in PubMed, retrieved 20.  
 

 What does Google say? 
 PDF only 
| .edu only 

---

**Interactive relationship** (e.g. stimulation, inhibition, etc)

**Neutral relationship**- Furthermore, overproduction or loss of Rga1 and Rga2, but not Bem3, affected the two hybrid interaction of  **Cdc42**  with Ste20, a p21  [ **PAK3** ]  activated kinase kinase required for haploid invasive growth.  Ref: 12455995 Eukaryot Cell, 2002
- The p21  [ **PAK3** ]  activated protein kinases are regulated by the Rho family GTPases Rac and  **Cdc42** .  Ref: 12529371 J Biol Chem< MedlineTA>J Biol Chem, 2002
- p21  [ **PAK3** ]  activated kinase 1 PAK1 is an effector of Rac  **Cdc42**  GTPases that has been implicated in the regulation of cytoskeletal dynamics, proliferation, and cell survival signaling.  Ref: 12522133 J Biol Chem, 2003
- Two closely related p21  [ **PAK3** ]  activated kinases from Saccharomyces cerevisiae, Ste20 and Cla4, interact with and are regulated by  **Cdc42** , a small Rho like GTPase.  Ref: 12588977 Mol Cell Biol, 2003
- p21  [ **PAK3** ]  activated protein kinases PAK are a family of serine threonine protein kinases which are activated by binding of the p21  [ **PAK3** ]  G proteins  **Cdc42**  or Rac.  Ref: 12853446 J Biol Chem< MedlineTA>J Biol Chem, 2003
- The p21  [ **PAK3** ]  activated protein kinases Paks are serine threonine protein kinases activated by binding to Rho family small GTPases, Rac and  **Cdc42** .  Ref: 12860998 J Biol ChemJ Biol Chem, 2003

**Non-interactive relationship** (e.g. studied together, co-existance, homology, etc.)

- Mutation of histidine residues 19 and 22 to leucine on the p21  [ **PAK3** ]  binding domain of Pak5 completely abolished the binding of  **Cdc42**  and the  **Cdc42**  mediated autophosphorylation.  Ref: 12860998 J Biol ChemJ Biol Chem, 2003
- In brain, Shank forms a complex with beta PIX and beta PIX associated signaling molecules including p21  [ **PAK3** ]  associated kinase PAK, an effector kinase of Rac1  **Cdc42** .  Ref: 12626503 J Biol Chem, 2003
- The p21  [ **PAK3** ]  activated kinases PAKs 1 3 are serine threonine protein kinases whose activity is stimulated by the binding of active Rac and  **Cdc42**  GTPases.  Ref: 12676796 Annu Rev Biochem< MedlineTA>Annu Rev Biochem, 2003
- The pathway initiated by m2 receptors involved sequential activation of Gbetagamma i3, phosphatidylinositol PI 3 kinase,  **Cdc42** , and Rac1, p21  [ **PAK3** ]  activated kinase PAK1, p38 mitogen activated protein MAP kinase, and cPLA 2, and phosphorylation of cPLA 2 at Ser 505.  Ref: 12576304 Am J Physiol Gastrointest Liver Physiol, 2003
- We describe a signaling mechanism involving Gbetagamma, PAK associated guanine nucleotide exchange factor PIXalpha,  **Cdc42** , and p21  [ **PAK3** ]  activated kinase PAK 1.  Ref: 12887923 Cell, 2003
- ATX activated  **Cdc42**  and Rac1 and increased complex formation between these small G proteins and p21  [ **PAK3** ]  activated kinase PAK.  Ref: 12482591 FEBS Lett, 2002
- The p21  [ **PAK3** ]  activated kinase PAK homolog, Shk1, is a critical component of a multi functional Ras  **Cdc42**  PAK complex required for viability, polarized growth and cell shape, and sexual differentiation in the fission yeast, Schizosaccharomyces pombe.  Ref: 12764130 J Biol Chem< MedlineTA>J Biol Chem, 2002
